# Acute Hepatitis due to Garcinia Cambogia Extract, an Herbal Weight Loss Supplement

**DOI:** 10.1155/2018/9606171

**Published:** 2018-07-26

**Authors:** Akshay Sharma, Elisa Akagi, Aji Njie, Sachin Goyal, Camelia Arsene, Geetha Krishnamoorthy, Murray Ehrinpreis

**Affiliations:** ^1^Resident, Department of Internal Medicine, Sinai-Grace Hospital, Detroit Medical Center/Wayne State University, USA; ^2^Fellow, Department of Internal Medicine, Division of Gastroenterology, Detroit Medical Center/Wayne State University, USA; ^3^Clinical Research Director, Sinai-Grace Hospital; Associate Program Director, Transitional Medicine Residency Program, Sinai-Grace Hospital, Detroit Medical Center/Wayne State School of Medicine, Detroit, MI, USA; ^4^Associate Program Director, Department of Internal Medicine, Sinai-Grace Hospital, Detroit Medical Center/Wayne State University, USA; ^5^Program Director, Department of Internal Medicine, Division of Gastroenterology, Detroit Medical Center/Wayne State University, USA

## Abstract

The Drug Induced Liver Injury Network reports dietary supplements as one of the most important causes of drug induced hepatotoxicity, yet millions of people use these supplements without being aware of their potential life-threatening side effects. Garcinia cambogia (GC) extract is an herbal weight loss supplement, reported to cause fulminant hepatic failure. We present a case of a 57-year-old female with no previous history of liver disease, who presented with acute hepatitis due to GC extract taken for weight loss, which resolved after stopping it and got reaggravated on retaking it. Obtaining a history of herbal supplement use is critical in the evaluation of acute hepatitis.

## 1. Introduction

Fighting obesity can be challenging, and dietary supplements advertised as “slimming aids” are being widely used by people to manage weight loss [1]. Some of these supplements have potential life-threatening side effects. Extract of Garcinia cambogia (GC), a tropical fruit, is sold as a weight loss supplement. Hydroxycitric acid (HCA) is the active ingredient in GC that is associated with fat metabolism and weight loss. Hepatotoxicity due to GC extract has been reported, including cases of fulminant hepatic failure [1]. We present a case of acute hepatitis due to GC extract, which resolved after stopping the supplement and got reaggravated on retaking it.

## 2. Case Presentation

A 57-year-old female with no previous history of liver disease presented with abdominal pain and vomiting for one day. The abdominal pain was described as 7/10 in severity, nonradiating, and diffuse, but most intense in the right upper quadrant. She denied previously experiencing any similar pain. She denied fever or chills but reported 3 episodes of nonbloody, nonbilious emesis after the pain started. There was a history of heart failure with preserved ejection fraction. She had been taking vitamins A and D and an herbal supplement for weight loss but she denied the use of any prescription weight loss medications. She denied using alcohol, acetaminophen, or any illicit drugs. Her vital signs were normal. Physical examination was significant for diffuse abdominal tenderness without any rigidity or guarding. There was no hepatosplenomegaly or scleral icterus. Laboratory evaluation revealed an alanine aminotransferase (ALT) of 738 U/L [normal: 7-55 U/L], aspartate aminotransferase (AST) of 856 U/L [normal: 8-48 U/L], and an alkaline phosphatase of 80 U/L [normal: 45-115 U/L]. Her total bilirubin was 2.4 mg/dL [normal: 0.1-1.2 mg/dL] and direct bilirubin was 1.4 mg/dL [normal: 0-0.4 mg/dL]. International normalized ratio (INR) was 1.19 [normal: 0.8-1.1] and prothrombin time (PT) was 12.7 seconds [normal 11-13.5 seconds]. Testing for hepatitis A, hepatitis B, hepatitis C, hepatitis E, Herpes-Simplex virus, Ebstein-Barr virus, Parvovirus, and Cytomegalovirus was negative. She had normal vitamins A and D levels ruling out hypervitaminosis as the cause of hepatitis. She tested negative for alcohol and acetaminophen. Anti-smooth muscle antibody, anti-mitochondrial antibody, antinuclear antibody, and anti-liver kidney microsomal antibody were negative. An iron profile was normal. Abdominal ultrasound showed a normal liver with normal echotexture and no biliary ductal dilatation. When the herbal supplement was scrutinized, it was found to be 100% pure GC fruit rind extract. She had consumed two capsules daily as recommended for about one month. Each capsule had 1400 mg of GC extract. She was asked to stop taking the supplement. Her liver enzymes decreased significantly with an ALT of 396 *μ*/L and AST of 138 *μ*/L within three days with resolution of her abdominal symptoms. Her total and direct bilirubin came down to 0.6 mg/dL and 0.4 mg/dL, respectively. Her INR and PT also normalized. She had a normal ALT of 25 and AST of 12 in the one-month follow-up visit. Six months later, patient was found to have an elevated ALT of 301 and AST of 69. On interrogation, it was found that she had started taking the same supplement again in the desperate need to lose weight. The CIOMS/RUCAM scale, a scoring system (Table 1) that is used to establish the etiology of liver damage when drug induced liver damage is suspected, gave a score of 11 for our patient (Table 2)[2, 3]. The score classifies the drug as highly probable (score ≥ 9), probable (score 6-8), possible (score 3-5), unlikely (score 1-2), and excluded (score 0) as the cause of liver injury [2, 3]. This lead us to conclude that the etiology of the patient's hepatitis was GC extract.

## 3. Discussion

The Drug Induced Liver Injury Network reports dietary supplements as one of the most important causes of drug induced hepatotoxicity [4]. Yet millions of people use these supplements without being aware of their potential life-threatening side effects. GC is a tropical fruit grown in South East Asia used as a culinary agent due to its sharp, sour taste [5]. Numerous GC products for weight loss are on the market in spite of the reported possible toxicity associated with the regular use of these supplements [5]. The most important ingredient of GC is HCA which blocks adenosine triphosphatase citrate lyase, a catalyst for the conversion process of citrate to acetyl-coenzyme A, which plays a key role in fatty acid, cholesterol, and triglyceride syntheses [5, 6]. HCA was one of the main components of hydroxycut products, recalled after the Food and Drug Administration received reports of 23 cases of hepatotoxicity [7]. GC extract and HCA are proposed to cause weight loss by increasing satiety through regulation of serotonin levels, reducing lipogenesis, and upregulating fat oxidation [5]. A meta-analysis of 9 randomized controlled trials of GC extract for weight loss found that it leads to only a small short term weight loss and causes mainly mild gastrointestinal side effects (average dose of GC extract used was 1-3 g) [7]. Cases of acute liver failure progressing to fulminant hepatic failure requiring liver transplantation have been reported due to GC extract [1, 8]. In the cases of fulminant hepatic failure, biopsy showed significant necrosis and collapse of liver parenchymal architecture. The exact mechanism by which GC extract causes liver injury is still unclear but a recent study on mice demonstrated that it exacerbates steatohepatitis by increasing hepatic collagen accumulation, lipid peroxidation, and levels of proinflammatory cytokines like tumor necrosis factor-alpha and monocyte chemoattractant protein-1 [9]. It also caused an increase in mRNA level of superoxide dismutase and glutathione peroxidase responsible for oxidative stress [9]. Since the patient had been taking vitamins A and D, we looked up the literature but could not find any reports of their interaction with GC extract. As far as we are aware, there is no reported medical treatment for GC extract-induced hepatitis. Acute hepatitis seems to be a reversible side effect of GC extract, which resolves when it is stopped, as demonstrated by our case. Our case also depicts that once resolved, hepatitis can be reinduced by GC extract. There have been no such reports in the literature to the best of our knowledge. Early recognition and discontinuation of GC extract were associated with resolution of the GC extract-induced acute hepatitis and prevented the progression to fulminant hepatic failure. As physicians, it is our responsibility to be informed and educate our patients about all the possible side effects of any supplements that they may be taking. Whenever we come across acute hepatitis of unexplained etiology, obtaining a detailed history of herbal supplement use is of utmost importance.

## Figures and Tables

**Table 1:** CIOMS/RUCAM scale.

**Criteria**	**Score**
**1. Time from drug intake until reaction onset**	

5-90 days	+2

<5 or >90 days	+1

**2. Time from drug withdrawal until reaction onset**	

<15 days	+1

>15 days	0

**3. Alcohol risk**	

Present	+1

Absent	0

**4. Age risk factor**	

>55 years	+1

<55 years	0

**5. Course of reaction**	

>50% improvement within 8 days	+3

>50% improvement within 30 days	+2

Worsening or <50% improvement in 30 days	-1

**6. Concomitant therapy **	

Time to onset incompatible	0

Time to onset compatible but with unknown reaction	-1

Time to onset compatible but known reaction	-2

Role proved in the case	-3

None or information not available	0

**7. Exclusion of non-drug-related causes **	

Rule out	+2

“Possible” to “not investigated”	0

Probable	-3

**8. Previous information on hepatotoxicity **	

Reaction unknown	0

Reaction published but unlabeled	+1

Reaction labeled in the product's characteristics	+2

**9. Response to re-administration **	

Positive	+3

Compatible	+2

Negative	-2

Not available or not interpretable	0

Plasma concentration of drug known as toxic	+3

Validated laboratory test with high specificity, sensitivity, and predictive values	Positive: +3
	Negative: -3

**Table 2:** Calculation of CIOMS score in our patient gave a score of 11.

Time from drug intake until reaction onset	5 to 90 days [+2]
Time from drug withdrawal until reaction onset	≤15 days [+1]
Alcohol risk factor	Absent [0]
Age risk factor	≥55 years [+1]
Course of the reaction	>50% improvement 8 days [+3]
Concomitant therapy	Time to onset compatible and known reaction [-2]
Exclusion of non-drug-related causes	Rule out [+2]
Previous information on hepatotoxicity	Reaction published but unlabeled [+1]
Response to re-administration	Positive [+3]
